# Comparison of Prostate Biopsy Using Multiparametric Magnetic Resonance Imaging in Patients with Prostate Biopsy Indications

**DOI:** 10.5152/eurasianjmed.2022.20349

**Published:** 2022-02-01

**Authors:** Mehmet Sefa Altay, Fatih Özkaya, Şaban Oğuz Demirdöğen, Fevzi Bedir, Hüseyin Kocatürk, Ahmet Emre Cinislioğlu, Sinan Yilmaz, Turgut Yapanoğlu

**Affiliations:** 1 Department of Urology, Health Sciences University, Erzurum Regional Training and Research Hospital, Erzurum, Turkey; 2 Department of Urology, Atatürk University School of Medicine, Erzurum, Turkey; 3 Department of Public Health, Atatürk University School of Medicine, Erzurum, Turkey

**Keywords:** Prostate cancer, multiparametric MRI, TRUS-guided prostate biopsy

## Abstract

**Objective:** This article aims to evaluate the efficacy of multiparametric magnetic resonance imaging before standard tru-cut biopsy in making prostate cancer diagnosis.

**Materials and Methods:** A total of 160 patients with prostate biopsy indications were prospectively evaluated between May 2017 and October 2018. Multiparametric magnetic resonance imaging was taken after obtaining a written informed consent from all patients. Cognitive transrectal ultrasound-guided biopsy was performed based on multiparametric magnetic resonance imaging results. Standard tru-cut biopsy was included to reduce false-negative rate. Statistical analysis was performed using the Statistical Package for Social Sciences version 20.0 software.

**Results:** The mean age of the patients was 65.94 ± 7.90 (48-84) years. Around 19.37% of the patients had a specificity in the digital rectal exam. The mean prostate-specific antigen value of the patients with adenocarcinoma was 42.1 ng/mL and it was 10.2 ng/mL in patients with benign prostate hyperplasia. It was observed that the prostate-specific antigen values in prostatic adenocarcinomas were significantly higher than those in benign prostate hyperplasia (*P* < .001). The results of multiparametric magnetic resonance imaging and the biopsy were 100% similar in terms of zones in patients with adenocarcinoma. All of the biopsy results of the patients who were evaluated to have normal prostate tissue in multiparametric magnetic resonance imaging were evaluated as benign prostate hyperplasia; on the other hand, 13.6% of PI-RADS 2 lesions, 14% of PI-RADS 3 lesions, 31.8% of PI-RADS 4 lesions, and 85.7% of PI-RADS 5 lesions were determined to be adenocarcinoma. It was observed that the prevalence of adenocarcinoma increased as the risk elevated in multiparametric magnetic resonance imaging (*P* < .001).

**Conclusion:** Multiparametric magnetic resonance imaging evaluated by experienced radiologists may be instructive of urologists and reduce the need for unnecessary biopsies.

## Main Points

- Multiparametric magnetic resonance imaging (Mp-MRI) sensitivity is high in demonstrating prostatic lesions.
- Multiparametric magnetic resonance imaging is a good guide in transrectal ultrasound-guided prostate biopsy.
- In patients with prostate-specific antigen elevation, Mp-MRI is a good option before prostate biopsy.

## Introduction

Prostate cancer (PCa) is the most common type of cancer in males in the developed countries and it ranks second, after lung cancer, in mortality rate.^1,2^ With early diagnosis, the disease remains limited to the organ, and the success rate of treatment and recovery is higher. However, if the diagnosis is made late and the stage of the disease progresses, it leads to higher mortality, morbidity, and treatment costs. As the disease stage progresses, cancer-related survival rate decreases.^3,4^

The standard method for the diagnosis of PCas is a transrectal ultrasound (TRUS)-guided biopsy.^5^ Benign result of TRUS-guided biopsy does not eliminate the possibility of PCa.^3^

In TRUS-guided needle biopsies, the false-negative rate was reported to be between 12% and 28%.^6,7^ This led to advanced imaging methods and performing target-specific biopsies. Therefore, today, multiparametric magnetic resonance imaging (Mp-MRI) is used. For the evaluation of Mp-MRI, at least 2 functional MRI sequences are added to anatomical sequences.^8,9^ In 2012, European Society of Urogenital Radiology (ESUR) published “Prostate Imaging Reporting and Data System (PI-RADS)” to standardize the evaluation and reporting of MRI.^9^ However, the ESUR published second version (PI-RADS v2) through the end of 2014 as it was identified that there were many limitations after rapid improvements in clinical and research area.^10^ Prostate Imaging Reporting and Data System v2 has been defined as clinically significant PCa for standardization of Mp-MR and to work with pathologists. A clinically significant PCa can be defined as cancers with Gleason score of 7 and above (3 + 4, obvious but non-dominant Gleason component 4), 0.5 cc and above cancer volume, or extraprostatic extension.^11^ It was improved in 2019 and took the final form as PI-RADS v2.1.^12^ In PI-RADS v2, lesions are categorized from 1 to 5 and these categories indicate the likelihood of PCa.^10^ While PI-RADS 1 indicates very low PCa likelihood, PI-RADS categories 2, 3, 4, and 5 indicate low, intermediate, high, and very high likelihood, respectively.^10^

In the present study, we studied whether Mp-MRI could be guiding for a cognitive TRUS biopsy when taken before prostate biopsy.

## Materials and Methods

The study population included 160 patients with elevated prostate-specific antigen (PSA) level and/or specificity in the digital rectal exam (DRE). The study protocol was approved by the local Ethics of Atatürk University School of Medicine. The patients were prospectively evaluated between May 2017 and October 2018. A written informed consent was obtained from the patients presenting with lower urinary tract symptoms, and PSA screening and the DRE were performed. Multiparametric magnetic resonance imaging was taken before any process in patients with prostate biopsy indication. None of the patients included in the study had a previous prostate biopsy.

### Inclusion and Exclusion Criteria

Patients with serum PSA level of >50 ng/mL, fixed prostate in the DRE, patients who have had a previous prostate biopsy, and who do not want to participate in the study were not included in the study.

Patients who were indicated for prostate biopsy (PSA elevation and/or findings in DRE) and approved the study were included in the study.

### Multiparametric Magnetic Resonance Imaging and Biopsy Protocols

Multiparametric magnetic resonance imaging was obtained from the patients who were indicated for biopsy after obtaining consent. The results of Mp-MRI were evaluated using Magnetom Skyra 3T MRI system (Siemens Healthineers, Erlangen, Germany). Multiparametric magnetic resonance imaging images were independently interpreted by a single experienced radiologist.

Antibiotic prophylaxis (a single dose of trimethoprim/sulfamethoxazole) and periprostatic nerve blockage (1 mL/5 mg bupivacaine) were applied to the patients before biopsy. Individual biopsies were taken from each lesion described in Mp-MRI and these biopsy numbers were recorded. In addition, standard 14-quadrant tru-cut biopsy was added after biopsies were taken from the lesions described in Mp-MRI in order not to miss cancer. Generally, a single lesion was defined in Mp-MRI in the same prostate tissue. In different defined prostate tissue, different cores were taken and recorded. All biopsies were performed by a single urologist. In addition, the results of patients whose biopsy result was BPH were evaluated separately.

When the patients who were called for a checkup with the pathology report of the biopsy were diagnosed with ≥3-core high-grade prostatic intraepithelial neoplasia and/or atypical small acinar proliferation (ASAP), a 24-core saturation prostate biopsy was performed. No new viewing was made again.

In this study, the results of the biopsy and Mp-MRI PI-RADS v2 were compared. The results of Mp-MRI were reported as normal prostate tissue or lesions ranging from 1 to 5 of PI-RADS categories.

Patients included in the study were evaluated for BPH and PCa separately. Age, PSA, DRE, and prostate volumes of the patients were recorded. Biopsy results were compared according to Mp-MRI results. In terms of localization, the biopsy core side and the side of the lesion described in Mp-MRI were compared. Adenocarcinoma frequencies were evaluated with Mp_MRI PI-RADS classification. In addition, Gleason score ratios of patients with adenocarcinoma were evaluated.

### Statistical Analysis

Statistical analysis was performed using Statistical Package for Social Sciences version 20.0 software (IBM SPSS Corp.; Armonk, NY, USA). Categorical data were expressed in number and percentage, and numerical data were expressed in mean ± standard deviation (SD). The Kolmogorov–Smirnov test, the graphical method, and standardized z values estimated for the Skewness and Kurtosis coefficients were used to evaluate whether that data showed a normal distribution or not. The Mann–Whitney *U* test was used to study the distribution between 2 groups of continuous numerical variables which are not normally distributed and χ^²^ test was used to compare categorical variables. In statistical analysis, a *P*-value of <.05 was considered significant.

## Results

The mean age of the patients was 65.94 ± 7.90 (48-84) years. Of 160 patients who underwent a biopsy, 30% (n = 48) were diagnosed with adenocarcinoma and 70% (n = 112) were diagnosed with BPH. The mean age of the patients diagnosed with adenocarcinoma was 68.73 ± 7.55 (50-83) years and it was 64.75 ± 7.77 (48-84) in patients with BPH (Table 1).

In DRE, the specificity was 19.37% (n = 31) in all patients, 58.33% (n = 28) in patients with adenocarcinoma, and 2.67% (n=3) in patients diagnosed with BPH (Table 1).

The mean PSA of the patients with adenocarcinoma was 42.1 ng/mL, and it was 10.2 ng/mL in patients diagnosed with BPH. The mean PSA values were significantly higher in patients with prostatic adenocarcinoma compared to patients with BPH (*P* < .001).

Generally, a single lesion was described in Mp-MRI, and there was pathological concordance in regions with different lesions. In 14 quadrant biopsies, positive cors were found to match the lesion localization described in Mp-MRI. The lesion localization described in Mp-MRI and the localization of the cor from adenocarcinoma were the same in the patients. In patients with a result of adenocarcinoma in Mp-MRI and the biopsy, it was observed that there was a 100% similarity in terms of zone (Table 2).

**Figure 1.**
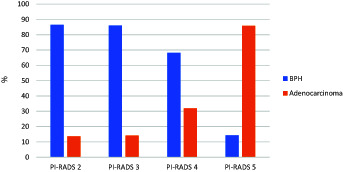
The distribution of Mp-MRI PI-RADS classification and the biopsy results. Mp-MRI, multiparametric magnetic resonance imaging; PI-RADS, prostate imaging reporting and data system.

The reports of the patients were indicated as normal prostate tissue or PI-RADS 2, 3, 4, and 5 regarding Mp-MRI PI-RADS v2. There was no report of PI-RADS 1. All of the biopsy results of the patients who were evaluated to have normal prostate tissue in Mp-MRI were evaluated as BPH. On the other hand, 13.6% of PI-RADS 2 lesions, 14% of PI-RADS 3 lesions, 31.8% of PI-RADS 4 lesions, and 85.7% of PI-RADS 5 lesions were determined as adenocarcinoma. The distributions of the results of Mp-MRI and the biopsies are shown in Table 3 and Figure 1.

When the distribution of biopsy results was evaluated regarding the PI-RADS risk classification of Mp-MRI, it was observed that there was a trend in Mp-MRI that increased the frequency of adenocarcinoma with PI-RADS risk classification and there was a significant difference between the distributions (*P* < .001, Table 3).

When pathological results of the patients with adenocarcinoma were compared in terms of Gleason scoring, a total of 58.3% of the patients had a Gleason score of 6. The distribution of Gleason scores of the patients with adenocarcinoma is shown in Table 4.

## Discussion

Prostate cancer is an important health problem in males. It is the most prevalent type of cancer in Europe and it ranks second among cancer types in terms of mortality rate.^2,13,14^ One of the important causes of PCa is age and the prevalence of the cancer increases with aging. It is suggested that the number of PCa patients will increase as the elderly population increases all over the world.^2,14,15^ The number of patients diagnosed with the cancer increased as PSA became available. This led to a decrease in the number of the cancer-related deaths and increases in the number of biopsies performed (70-80% more biopsies) and the treatment regimens administered.^4,16^ Therefore, there is a need for new methods to reduce the number of biopsies and prevent excessively administered treatments. Shah et al^17^ showed that the number of prostate biopsies decreased, but the rate of PCa diagnosis regarding the biopsies increased. In another study, it was reported that false-negative results in the biopsies were significant, being between 12% and 28%.^6^ Gittes et al^18^ identified that the rate of the determination of PCa in postmortem biopsies was between 15% and 70%. This led to the introduction of new imaging methods and further studies on reducing the false-negative rate and preventing unnecessary biopsies.^19^

Elevated PSA is not cancer-specific and it can increase in different types of prostate diseases. This situation leads to unnecessary number of biopsies performed. Heijnsdijk et al^16^ showed that 70-80% more biopsies were performed. In our study, BPH was diagnosed in 70% of the biopsies. This led to investigating new cancer-specific methods. Diagnostic accuracy and use of MRI gradually increases in the diagnosis of PCa together with the improvements in MRI techniques in recent years.^20^ Today, this is the most frequently used technique for prostate imaging.

In a study conducted by Choi et al^21^ with an upper PSA limit of 20 ng/mL, TRUS-guided biopsies were performed by urologists and MRI-guided biopsies were performed by radiologists. In TRUS-guided biopsies, the prevalence of PCa was 41.4% and it was 55.4% in MRI-guided biopsies. Moreover, it was also reported that the rates of the diagnosis of PCa were 8.3% in PI-RADS 3, 53.45 in PI-RADS 4, and 90.1% in PI-RADS 5. In a study conducted by Osses et al.^22^ including 155 patients, there was no cancer diagnosis in patients reported as PI-RADS 2; however, the rates of cancer diagnosis were 10% in PI-RADS 3, 77% in PI-RADS 4, and 89% in PI-RADS 5. It was also reported that 63% of the patients had a Gleason score of ≥7. In our study, the rates of adenocarcinoma diagnosis were 13.6% in PI-RADS 2, 14% in PI-RADS 3, 31.8% in PI-RADS 4, and 85.7% PI-RADS 5. Of the patients who underwent a biopsy, 30% were diagnosed with PCa and 41.7% had a Gleason score of ≥7. We consider that the differences between the studies result from the radiologists who evaluated Mp-MRIs.

Kuru et al^23^ reported a negative predictive value of 99% for the lesions reported to be PI-RADS 2-3. They also suggested that positive predictive value was 83% for PI-RADS 4 and 5. On the other hand, in our study, negative predictive value was 100% and positive predictive values for PI-RADS 4 and PI-RADS 5 were 31.8% and 85.7%, respectively.

Ahmed et al^24^ used 1.5 T MRI in their study and showed that Mp-MRI reduced biopsy rates by 25%. Moreover, it was also suggested that with the use of Mp-MRI, the rate of the diagnosis of clinically significant PCa increased while that of clinically insignificant PCa decreased. In our study, 3 T MRI was used and we consider that Mp-MRI helps reduce unnecessary biopsies even though the focus was not on clinically significant PCa.

Bass et al^25^ took Mp-MRI before a biopsy and accordingly performed biopsies on the patients. They showed that the rate of the diagnosis of clinically significant PCa increased in patients whose Mp-MRIs were taken. On the other hand, although the focus was not on clinically significant PCa, our study results showed that taking Mp-MRI before a biopsy may increase the rate of the diagnosis of adenocarcinoma in biopsy and reduce the number of unnecessary biopsies.

Popita et al^26^ indicated that PCa could be eliminated via Mp-MRI with high precision. In that study, it was also suggested to take Mp-MRI before performing a biopsy. That the results of the pathologies of all patients with a normal Mp-MRI result were benign supports this idea.

Kam et al^27^ reported that in patients with PI-RADS score of ≥3 in Mp-MRI, clinically significant PCa was at a susceptibility rate of 91% with a positive predictive value of 95%. The mean PSA was estimated to be 9.5 ng/mL. Clinically significant PCa was diagnosed in 10% of the patients reported as PI-RADS 1 or 2, 16% of the patients reported as PI-RADS 3, and 74% of the patients reported as PI-RADS 4 or 5. The difference in our study was that the focus was not on clinically significant cancer diagnosis. As shown in Table 3, there are some differences in PI-RADS scores and cancer rates compared to that study.

In a study conducted by Bryant et al^28^ on the comparison of the patients who underwent a biopsy previously with or without Mp-MRI taken, there was no increase in the number of PCa diagnosis, Gleason scores, and positive core values. There was also a significant correlation between PI-RADS score and the number of malign biopsies. Moreover, it was reported that clinically significant PCa at a rate of 5-15% could be missed without a biopsy when PI-RADS score was 1-2. On the other hand, our study results suggested that the prevalence of cancer increased with the increase in PI-RADS score and the relation was statistically significant, although the focus was not on clinically significant PCa. That a total of 13.6% of the patients reported as PI-RADS 2 were diagnosed with the cancer supports the idea that cancer may be missed if a biopsy is not performed.

Tonttila et al^29^ compared TRUS-guided biopsies and the patients who underwent fusion-guided biopsy and identified that there were similar rates of diagnosis of PCa and clinically significant PCa. In the present study, we performed TRUS-guided cognitive biopsy under the guidance of the reports of Mp-MRI taken before the biopsy. We consider that taking Mp-MRI before a biopsy in settings where fusion-guided biopsy cannot be performed may provide guidance to TRUS-guided biopsies.

Our study is similar to the literature in terms of its results. Biopsy of the lesion with cognitive biopsy after lesion localization with Mp-MRI is satisfactory in terms of results. Multiparametric magnetic resonance imaging before TRUS-biopsy seems to be a good option for urology clinics without fusion biopsy. However, it is obvious that studies with large series of patients are needed.

The limitation of the study was the relatively small number of patients and the fact that it was a single-center study. However, the advantage of the study was that Mp-MRI was evaluated by a single radiologist, TRUS biopsy was performed by a single urologist, and it was a prospective study.

In conclusion, the idea that biopsies should be avoided when Mp-MRI is reported to be normal due to elevated negative predictive value comes into prominence. Low-grade lesions can be followed up in Mp-MRI and it may be decided upon a biopsy accordingly. Especially in patients whose biopsies are contraindicated and who carry risk for biopsies due to comorbidities or who does not accept biopsies, it should be considered that Mp-MRI is an important option before a prostate biopsy and the decision may be made regarding the grade of lesions.

In conclusion, PCa is an important public health problem. Accurate diagnosis should be made and unnecessary processes should be avoided. If the rates of PCa diagnosis can be increased, unnecessary processes can be avoided via Mp-MRI evaluated by experienced radiologists. The diagnosis, treatment, and follow-up of PCa may be more effective through getting Mp-MRI into a routine in clinical practices, especially with the cooperation of radiologists and urologists.

## Figures and Tables

**Table 1.** Demographical/Physical Examination Results and the Mean PSA and Prostate Volumes of the Patients

Variables	Adenocarcinoma	BPH	Total	*P*
Number of the patients [% (n)]	30 (48)	70 (112)	100 (160)	
Mean age (year)	68.73 ± 7.55	64.75 ± 7.77	65.94 ± 7.90	.003
DRE positive [% (n)]	58.33 (28)	2.67 (3)	19.37 (31)	<.001
PSA (ng/mL)	42.16 ± 59.69	10.25 ± 10.38	19.82 ± 36.65	<.001
Prostate volumes (mL)	68.87 ± 28.30	83.23 ± 47.89	78.92 ± 43.38	.109

DRE, digital rectal exam; PSA, prostate-specific antigen; BPH, benign prostate hyperplasia.

**Table 2.** Distribution of Mp-MRI Results of the Patients with a Result of Cancer in the Biopsy

Variables	Transitional Zone	Right Peripheral Zone	Left Peripheral Zone	Bilateral	Total
No. of the patients with biopsy [% (n)]	4.16 (2)	41.66 (20)	45.83 (22)	8.33(4)	100 (48)
No. of the patients with Mp-MRI [% (n)]	4.16 (2)	41.66 (20)	45.83 (22)	8.33(4)	100 (48)

Mp-MRI, multiparametric magnetic resonance imaging.

**Table 3.** The Distributions of the Results of Mp-MRI and Prostate Biopsies

Mp-MRI	Adenocarcinoma [% (n)]	BPH [% (n)]	Total [% (n)]	*P*
No specificity	-	100.0 (16)	100.0 (16)	.001
PI-RADS 2	13.6 (3)	86.4 (19)	100.0 (22)	
PI-RADS 3	14.0 (7)	86.0 (43)	100.0 (50)	
PI-RADS 4	31.8 (14)	68.2 (30)	100.0 (44)	
PI-RADS 5	85.7 (24)	14.3 (4)	100.0 (28)

Mp-MRI, multiparametric magnetic resonance imaging; PI-RADS, prostate imaging reporting and data system; BPH, benign prostate hyperplasia.

**Table 4.** The Distribution of Gleason Scores of the Patients with Adenocarcinoma

Gleason Score	% (n)
3 + 3 = 6	58.3 (28)
3 + 4 = 7	10.4 (5)
4 + 3 = 7	6.3 (3)
4 +4 = 8	2.1 (1)
3 + 5 = 8	2.1 (1)
4 + 5 = 9	4.2 (2)
5 + 4 = 9	8.3 (4)
5 + 5 = 10	8.3 (4)
Total	100.0 (48)
